# *Tsukamurella tyrosinosolvens* bacteremia with coinfection of *Mycobacterium bovis* pneumonia: case report and literature review

**DOI:** 10.1186/s40064-016-3707-y

**Published:** 2016-11-29

**Authors:** Chang-Hung Chen, Chao-Tai Lee, Tsung-Chain Chang

**Affiliations:** 1 Department of Internal Medicine, Chest Division, Tainan Municipal Hospital, Tainan, Taiwan; 2 Department of Clinical Laboratory, Tainan Municipal Hospital, Tainan, Taiwan; 3 Department of Medical Laboratory Science and Biotechnology, College of Medicine, National Cheng Kung University, Tainan, Taiwan

**Keywords:** Pneumonia, Bacteremia, PCR (polymerase chain reaction), rRNA (ribosome RNA)

## Abstract

**Introduction:**

We describe an immunocompromised patient with *Tsukamurella tyrosinosolvens* bacteremia and coinfection of *Mycobacterium bovis* pneumonia.

**Case Description:**

A 75-year-old male was admitted to our hospital complaining of persistent fever with general malaise. His medical history showed that he had diabetes mellitus (HbA1C 9.2%). A chest computed tomography (CT) showed left upper lung consolidation . Two sets of blood culture at admission finally showed *Tsukamurella tyrosinosolvens*. Moreover, three transbronchoscopy washing specimen cultures revealed *Mycobacterium bovis*.

**Discussion and Evaluation:**

The organism *Tsukamurella tyrosinosolvens* was identified using conventional biochemical identification methods, PCR-restriction DNA fragment analysis, and 16S rRNA gene sequencing. The clinical mycobacterial isolates were identified to the species level by combining Polymerase Chain Reaction (PCR) with an oligonucleotide microarray to detect the *M. bovis* amplicons.

**Conclusion:**

According to our literature review, our patient’s case was the first of a coinfection with *Tsukamurella tyrosinosolvens *and *Mycobacterium bovis*. Prolonged antibiotic treatment and underlying disease control are necessary for this type of patient.

## Case report

**Fig. 1:**
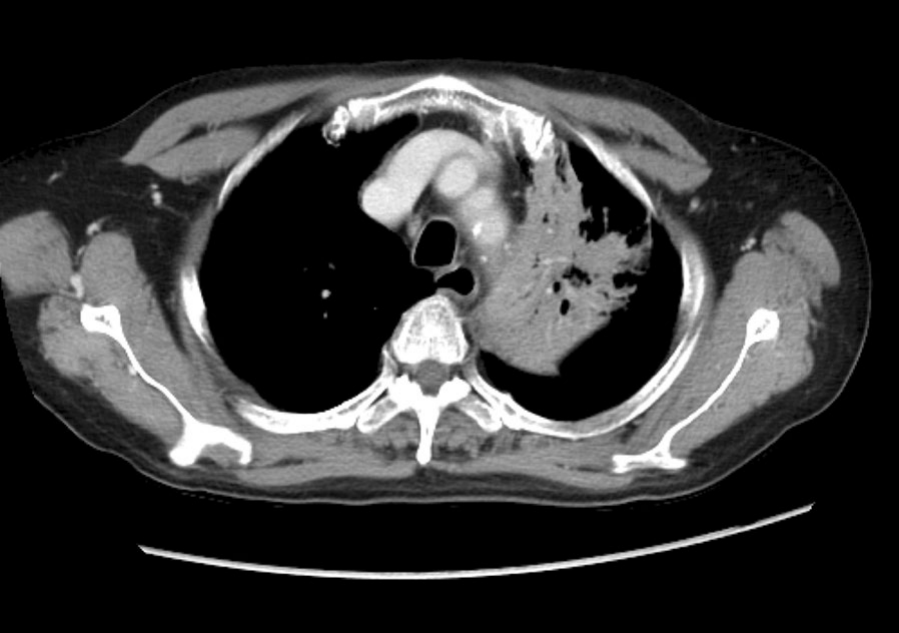
The chest CT shows left upper lung consolidation

A 75-year-old male was admitted to our hospital complaining of persistent fever with general malaise for approximately 1 week. The patient had a productive cough and his medical history showed that he had diabetes mellitus (HbA1C 9.2%), hypertensive cardiovascular disease, and chronic obstructive pulmonary disease. He used oral medication and an inhaled bronchodilator to control his aforementioned diseases. In the admission survey, a complete blood count revealed that the patient had leukocytosis (WBC 21,000 μL). Moreover, a biochemistry survey showed that he had an elevated C-reactive protein level (20.3 mg/dl). A chest X-ray revealed that his left upper lobe was consolidated. The blood culture and sputum culture were performed at the admission under the impression of pneumonia. First, intravenous third-generation cephalosporin (ceftriaxone) combined with macrolide (azithromycin) were used. The patient’s clinical symptoms improved slowly, but he developed a high fever. A chest computed tomography (CT) performed 1 week later still showed left upper lung consolidation (Fig. 1). The bacteria laboratory then reported gram-positive bacilli growth in the patient’s blood culture, and the acid-fast stain of the bacteria in blood specimen was also revealed to be positive. *Nocardia* spp. was highly suspected. We performed a bronchoscopy for the lung lesion, and multiple white cheese-like plaque coatings were observed over the left upper lobe bronchial orifice. The transbronchoscopy washing, brushing, and biopsy were performed. The sputum specimen direct smear showed acid-fast stain positive bacilli, and a biopsy specimen showed granulomatous inflammation. According to the blood culture data and bronchoscopy results, a *Nocardia* spp. pulmonary infection with sepsis was highly suspected. Hence, we changed the antibiotics to intravenous carbapenum (imipenem) and sulfonamide trimethoprim. The patient’s clinical condition improved gradually. After 2 weeks, two sets of blood culture at admission finally showed *Tsukamurella tyrosinosolvens.* Moreover, three transbronchoscopy washing specimen cultures revealed *Mycobacterium bovis* 1 week later. We started isoniazid, rifabutin, and ethambutol treatment for the lung lesion immediately. The use of imipenem was 3 weeks and the sulfonamide and trimethoprim treatment was maintained for 1 month. Furthermore, the antituberculosis treatment was maintained for 12 months. After medical management, the patient’s chest X-ray survey and physical condition markedly improved.

## Microbiology and methods

Gram-positive, weakly acid-fast bacilli were isolated from blood cultures (Bactec FX system, Becton–Dickinson and Company, Sparks, MD). The isolate grew effectively at 37 °C on a blood agar plate. The colonies were 2–5 mm in diameter, dry, rough, branched, and had a slightly yellowish pigment appearance. The biochemical profile of the isolate was catalase-positive and urease-positive, and it hydrolyzed tyrosine but could not hydrolyze xanthine or casein in a *Nocardia* agar plate (Creative Media Products Ltd, New Taipei City, Taiwan).

Genotypic identification was performed using 16S rRNA gene sequencing. The 16S rRNA gene was amplified by primers 11F (5′-GTTTG ATCCTGGCTCAG-3′) and 1512R (5′-GGYTACCTTGTTACG ACTT-3′). Amplification was executed in an Applied Biosystems 2720 thermal cycler (Applied Biosystems, Taipei, Taiwan). The PCR reaction mixture (25 μL) contained 2.5 μL of genomic DNA, 1× KAPA 2G Fast HotStart ReadyMix, and 1 μM forward and reverse primers. After an initial denaturation at 95 °C for 3 min, 10 cycles of amplification (95 °C for 15 s and 60 °C for 50 s) and 36 cycles of amplification (95 °C for 15 s, 55 °C for 30 s, and 72 °C for 20 s) were conducted. The amplicon was purified and sequenced. The sequences obtained (1046 bp) were matched to that of *Tsukamurella tyrosinosolvens* present in the GenBank database by using the BLAST algorithm (http://www.ncbi.nlm.nih.gov/blast).

The transbronchoscopic washing specimens were decontaminated using *N*-acetyl-l-cysteine (NaLC) (0.5% final concentration)–NaOH (2% final concentration), and concentration by centrifugation was performed. Part of the sediment was used for the acid fast smear, which was then stained using auramine-rhodamine fluorochrome staining. Part of the NaLC–NaOH sediment or sterile specimen was inoculated onto a Löwenstein–Jensen solid medium and into a 7H12 broth liquid medium that was monitored for growth by using the Bactec 960 instrument.

The BACTEC™ MGIT™ 960 (Becton–Dickinson and Company) is a liquid broth media useful for recovering mycobacteria from clinical samples. In this study, samples were digested and decontaminated using the standard sodium hydroxide NaLC–NaOH method. Once a positive signal was provided by the BACTEC system, a Ziehl–Neelsen staining was performed, and the presence or absence of serpentine cording morphology was observed. The clinical mycobacterial isolates were identified to the species level by combining Polymerase Chain Reaction (PCR) with an oligonucleotide microarray to detect the *M. bovis* and *M. tuberculosis* amplicons.

## Discussion

*Tsukamurella* species are categorized to the order *Actinomycetales.* They are obligate aerobic, gram-positive, partially acid-fast, nonmotile bacilli (Schwartz et al. 2002). *Tsukamurella* bacteria have many features very similar with other *Actinomycetales*, such as *Nocardia*, *Rhodococcus*, *Gordonia*, and rapidly growing *Mycobacterium* bacteria. Using the standard biochemical tests, *Tsukamurella* species might be misidentified as belonging to one of these genera (Alcaide et al. 2004). Infections of *Tsukamurella* species mostly relate to an immunocompromised condition (malignancy, postchemotherapy, AIDS, and chronic renal failure) and the use of intravascular catheters and prosthetic devices (Schwartz et al. 2002; Alcaide et al. 2004; Granel et al. 1996; Larkin et al. 1999). Clinical syndromes in reported cases of *Tsukamurella* infection include bacteremia (Schwartz et al. 2002), cutaneous infection (Schwartz et al. 2002), lung infection (Alcaide et al. 2004), conjunctivitis (Elshibly et al. 2005), infection of a knee prosthesis and defibrillator (Granel et al. 1996; Larkin et al. 1999), and brain abscess (Sheng et al. 2009).

Accurately identifying *Tsukamurella* spp. by using phenotypic methods is difficult. The underreporting of *Tsukamurella* spp. resulted from the similarity of this genus to other more common pathogens expected in immunocompromised patients, such as *Mycobacterium* (Stanley et al. 2006). The culture-medium morphology, slow growth, and weak acid-alcohol-fastness characteristics of *Tsukamurella*, will cause misdiagnoses of *Corynebacterium, Rhodococcus, Nocardia, Gordonia*, or *Mycobacterium* species infections. A PCR-restriction fragment-length polymorphism analysis of hsp65 has been reported to be useful in identifying all clinically crucial species of aerobic actinomycetes (Steingrube et al. 1997). To identify atypical pathogens, 16S rRNA sequencing is a relatively rapid and reliable new molecular technique (Sheridan et al. 2005).

Optimal treatment of infections caused by *Tsukamurella* spp. is undetermined, mainly due to the rarity of infections caused by these pathogens and lack of standard susceptibility methods for *Tsukamurella* spp. The previous susceptibility reports have shown that *Tsukamurella* isolates were susceptible to aminoglycosides, macrolides, fusidic acid, imipenem, ciprofloxacin, trimethoprim and sulfamethoxazole, vancomycin, and teicoplanin, but resistant to penicillin, oxacillin, tetracycline, chloramphenicol, and expanded-spectrum cephalosporin (Liu et al. 2011).

To treat our patient with *T. tyrosinosolvens* sepsis, we used imipenem combined with trimethoprim and sulfamethoxazole. The optimal length of treatment for infections caused by *Tsukamurella* spp. bacteria has not been determined and should be individualized according to clinical response. We maintained the treatment of our patient with *T. tyrosinosolvens* sepsis until the patient’s clinical symptoms markedly improved.

Human tuberculosis is caused principally by *Mycobacterium tuberculosis*. The main causative agents of bovine tuberculosis is *Mycobacterium bovis* and cattle are the major reservoir. The zoonotic transmission of this pathogens occurs primarily through close contact with infected cattle or consumption of contaminated animal products such as unpasteurized milk. Tuberculosis cases caused by transmission of mycobacteria from animal reservoirs (e.g., wildlife) have been anecdotally reported. Globally, most cases of zoonotic tuberculosis are caused by *M. bovis* (Müller et al. 2013).

*Mycobacterium bovis* is intrinsically resistant to pyrazinamide (PZA). The appropriate treatment for *M. bovis* is extrapolated from experience in treating PZA-resistant *M. tuberculosis*. For otherwise sensitive pulmonary and extrapulmonary *M. bovis*, treatment consists of 2 months of isoniazid, rifampin, and ethambutol (administered daily), followed by 7 months of isoniazid and rifampin (LoBue and Moser 2005). The duration of therapy for pulmonary and most extrapulmonary disease should be as least 9 months. For our patient, considering his immunocompromised state (diabetes HbA1C 9.2 and combined *T. tyrosinosolvens* bacteremia), we opted to maintain medical treatment for 12 months.

At the beginning of hospitalization, the initial acid-fast-stain-positive bacteria result prompted us to conclude that the pathogens in pneumonia and blood were the same. Two sets of blood culture revealed *T. tyrosinosolvens* made us to believe the result was not related to contamination of the culture process. And the transbronchoscopy washing specimen culture did not reveal *T. tyrosinosolvens* after careful examination and incubation. After examining our patient’s medical history, we found that his son was a beef merchant, and our patient worked in the traditional Taiwanese market. Our patient did not have well diabetes control so the possible route of infection in our patient was environmental exposure through food consumption in an immunocompromised status. After discussing with our patient, he agreed and gave his consent to us for the use of his personal and medical information for the publication of this case report and any accompanying images.

## Conclusion

According to our literature review, our patient’s case was the first of a coinfection with *T. tyrosinosolvens* and *M. bovis*. The similarity of these 2 types of organisms on the basis of conventional identification methods lead doctors to treat infections with these organisms incorrectly. Careful and accurate identification of the bacteria causing an infection is necessary for patients with infections similar to that of our patient. Prolonged antibiotic treatment and underlying disease control are also necessary for this type of patient.

## Abbreviations

PCR: polymerase chain reaction
rRNA: ribosomal RNA

## References

[CR1] Alcaide ML, Espinoza L, Abbo L (2004). Cavitary pneumonia secondary to Tsukamurella in an AIDS patient. First case and a review of the literature. J Infect.

[CR2] Elshibly S (2005). Central line-related bacteraemia due to *Tsukamurella tyrosinosolvens* in a haematology patient. Ulster Med J.

[CR3] Granel F (1996). Cutaneous infection caused by *Tsukamurella paurometabolum*. Clin Infect Dis.

[CR4] Larkin JA (1999). Infection of a knee prosthesis with *Tsukamurella* species. South Med J.

[CR5] Liu C-Y (2011). Clinical characteristics of infections caused by *Tsukamurella* spp. and antimicrobial susceptibilities of the isolates. Int J Antimicrob Agents.

[CR6] LoBue PA, Moser KS (2005). Treatment of *Mycobacterium bovis* infected tuberculosis patients: San Diego County, California, United States, 1994–2003. Int J Tuberc Lung Dis.

[CR7] Müller B (2013). Zoonotic *Mycobacterium bovis*-induced tuberculosis in humans. Emerg Infect Dis.

[CR8] Schwartz MA (2002). Central venous catheter-related bacteremia due to *Tsukamurella* species in the immunocompromised host: a case series and review of the literature. Clin Infect Dis.

[CR9] Sheng W-H (2009). Brain abscess caused by *Tsukamurella tyrosinosolvens* in an immunocompetent patient. J Clin Microbiol.

[CR10] Sheridan EAS (2005). *Tsukamurella tyrosinosolvens* intravascular catheter infection identified using 16S ribosomal DNA sequencing. Clin Infect Dis.

[CR11] Stanley T (2006). The potential misidentification of *Tsukamurella pulmonis* as an atypical Mycobacterium species: a cautionary tale. J Med Microbiol.

[CR12] Steingrube VA (1997). Rapid identification of clinically significant species and taxa of aerobic actinomycetes, including Actinomadura, Gordona, Nocardia, Rhodococcus, Streptomyces, and Tsukamurella isolates, by DNA amplification and restriction endonuclease analysis. J Clin Microbiol.

